# Influence of Inclination of Welding Torch on Weld Bead during Pulsed-GMAW Process

**DOI:** 10.3390/ma13112652

**Published:** 2020-06-10

**Authors:** Ping Yao, Heqing Tang, Kang Zhou, Hongyan Lin, Zihui Xu, Xiongzi Du

**Affiliations:** 1 College of Electromechanical Engineering, Guangdong Polytechnic Normal University, Guangzhou 510635, China; gsyaop@gpnu.edu.cn (P.Y.); happythq2015@163.com (H.T.); sjy843644862@163.com (H.L.); xzh110592@163.com (Z.X.); 2 School of Mechatronical Engineering, Beijing Institute of Technology, Beijing 100081, China; 3120190179@bit.edu.cn

**Keywords:** arc welding process, inclination, weld bead, curve fitting, penetration

## Abstract

This work is about the influence rule of inclination of welding torch on the formation and characteristics of weld bead during the pulsed-gas metal arc welding (GMAW) process based on the robotic operation. The inclination of welding torch was an important operation condition during the pulsed-GMAW process, because it can affect the formation and quality of weld bead, which was the output of the process. In this work, the different inclination modes and values were employed to conduct actual welding experiments, and some influence rules can be obtained according to examine the surface topography and cross section. Then, to obtain further rules, serious measurements for the geometry characteristic parameters were conducted and corresponding curve fitting equations between inclination angles and the bead width, penetration and bead height were obtained, and the largest error of these curve fitting equations was 0.117 mm, whose corresponding mean squared error (MSE) was 0.0103. Corresponding verification experiments validated the effectiveness of the curve fittings and showed the second order polynomials were proper, and the largest errors between measurements and curve fitting equations for inclination angle under backward mode were larger than those under forward mode, and were 0.10 mm and 0.15 mm, respectively, which corresponded to the penetration and were below 10%, therefore the equations can be used to predict the geometry of the weld bead. This work can benefit the process and operation optimization of the pulsed-GMAW process, both in the academic researches and actual industrial production.

## 1. Introduction

Pulsed-gas metal arc welding (GMAW) is a commonly employed arc welding process which was employed in industrial metal joining process and other relative areas [1,2,3]. In this process, the pulsed welding current generated from the welding power source is used to control the metal droplet included in the electrical arc. Using this process, stable electrical arc can be induced even various welding parameters are so small. Because the process can accurately control the heat energy of the electrical arc, the pulsed-GMAW process is more and more commonly employed for joining types of base metals [4]. Compared to the conventional GMAW process, the pulsed-GMAW process has various advantages, such as high productivity and process robustness, and obtains the products with fine grain size [5]. It can not only adjust the value and duration of base current to decrease the heat delivery, so as to avoid very large deformation and burn-off the base metals, but can also utilize the high peak current to realize the desired one-droplet-per-pulse (ODPP) metal transfer mode. The metal transfer mode can directly affect the formation and the surface of the weld bead [6]. The various merits of this technology make it being increasingly used for joining a wide variety of industrial occasions, due to its inherit advantages such as deep penetration, smooth weld bead, high welding speed, large metal deposition rate, lower spatter, lower distortion and shrinkage, lesser probability of porosity and fusion defects, and controllable heat input and all-position welding [7,8,9]. It is an advanced spray transfer process with low mean current, and the welding current is pulsed between high and low levels of short or long time intervals so that it brings the weld zone to the melting point during the pulse current period and allows the molten weld pool to cool and solidify during the background current period [7]. Hence, this process can realize a stable and controllable metal transfer process, and obtain weld beads with desired surface topography. 

The evaluation of the welding quality is so important for process improvement and product optimization. Different welding processes had different evaluation criteria. For example, the nugget size or tensile-shear strength can be used to evaluate the quality of resistance spot welding [10,11]. During the GMAW process, the formation and characteristic of weld bead is the most commonly employed criterion to evaluate the quality of the operation, and this criterion involves more elements, such as crack, appearance, geometry characteristics, microstructure, and so on [12].

To meet the huge requirements of the actual industrial applications, many scholars and experts took efforts to explore the influences of the different operational conditions on the formation and characteristic of weld bead. Some relative contributions have been reported in the past decades. Rodrigues et al. [13] investigated the influences of three kinds of shielding gases and two activating fluxes on the geometry of welds produced by the tungsten inert gas (TIG) welding processes, which was other commonly used arc welding process. Shoeb et al. [14] studied the effects of some process parameters, such as welding speed, voltage and gas flow rate on the weld bead geometry, such as penetration, width and height, and in the work, mathematical equations were developed to describe the relations between the parameters and geometry parameters using factorial technique. In addition, to explore the weld bead forming rule during the double-pulsed GMAW process, our research group [15] employed the grey relational analysis method to quantitative establish the relations between some key operational parameters and the geometry characteristic parameters of the weld bead. The results showed that the average welding current and welding speed were the key elements which affected the characteristic parameters of the weld bead. Additionally, the same group [16] studied the effects of the operational parameters on the ripples of the weld bead; corresponding analyses showed that the most influential element on the distances of ripples was the welding speed, and the following was the twin pulse frequency. Moreover, recently, to increase the productivity during the actual production, the GMAW process usually collaborated with the industrial robot operation, which can significantly improve the accuracy of the real time control and make the operations more and more convenient. Aviles-Viñas et al. [17] proposed a real time computer vision algorithm to extract training patterns in order to acquire knowledge for predicting specific geometries, and the proposal was implemented and tested by an industrial KUKA robot and a GMAW type machine within a manufacturing cell. Chen et al. [18] employed a welding robot to acquire and optimize the weld trajectory and pose information, based on a laser sensor, charge-coupled device and other auxiliary instrument. Yang et al. [19] used an arc welding robot to detect the welding quality based on three-dimensional reconstruction using a special algorithm, and the results showed the system can quickly and efficiently fulfill the detection task of welding quality. These works denoted that using an industrial robot combined other technologies, many tasks which cannot be accomplished by traditional operations can be realized.

Despite that many reported contributions concerned that the influences of different operational parameters on the quality of weld bead, the relative works were mainly about the welding current, robot welding speed, and other common real-time control parameters. However, as a special and important operational condition, the inclination of the welding torch can also affect the formation and surface topography of the weld bead, because the welding torch is an executive component which is directly related to the energy delivery from the power source to the objective base metal. In general, the inclination of the welding torch was difficult to accurately adjust in the conventional GMAW operation because relative reported contributions were so few. Recently, as there has been fast improvement of the welding process based on robotic operation, online controlling of the inclination can achieve a high accuracy. Hence, in this work, an industrial robot was employed to accurately control the inclination and detailed influence rule of the different modes and values on the formation and characteristics of the weld bead during the pulsed-GMAW process can be seriously examined, in order to improve the GMAW process and obtain weld bead with high quality. To obtain the detailed influence rule, corresponding curve fittings between inclination angles and geometry characteristic parameters were also conducted. Corresponding verification experiments validated the accuracy of the curve fittings. The work is supposed to serve the academic researches of this type of metal joining technology, and promote the process improvement in actual industrial production. 

## 2. Experimental Platform and Procedures

### 2.1. Experimental Platform

**Figure 1:**
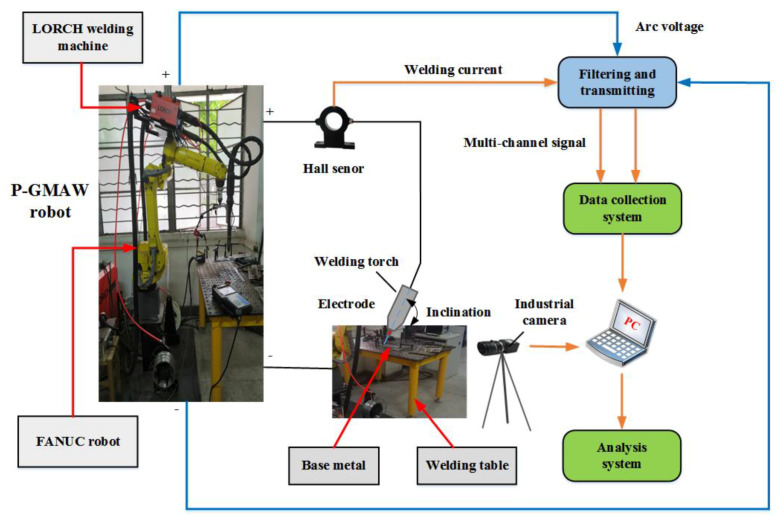
Structure and platform of the experiment.

To obtain reliable results, a FANUC Robot M-10iA industrial robot (FANUS Corporation, Oshino-mura, Yamanashi Prefecture, Japan) was incorporated to precisely control welding speed and the inclination of the welding torch. Apart from being an industrial robot, a LORCH S-RobotMIG (Lorch Schweißtechnik GmbH, Im Anwänder, Auenwald, Germany) acts an arc welding machine, a wire feeder, a welding torch, and an industrial camera, and some relative other auxiliary equipment were also used. In addition, a self-designed multi-channel signal collection system and corresponding analysis system were used to collect arc voltage, welding current and other necessary electrical signals during the process. Figure 1 showed the experimental structure including the instruments used in this work.

### 2.2. Experimental Condition

The base metal in this experiment used the 304 stainless steel, and the corresponding tensile strength was 520 MPa and size of each specimen was 250 mm × 60 mm × 3 mm. The welding wire used was 316L stainless steel with a diameter of 1.2 mm, and the length of the stick-out of welding wire was maintained at 12 mm during the process. Table 1 showed the material characteristics of the base metal and welding wire.

In addition, the shielding gas was 98% pure argon and 2% CO_2_ with a flow rate of 15 L/min.

In the work, the welding operation was conducted using robotic means, so the controls and operations can be accurately executed. The welding speed in this experiment was fixed as 30 cm/min. To obtain reliable and convinced experimental results and eliminate noises during the experimental process, the surface of each base metal was processed using an angle grinder and then degreased with alcohol. The work used flat-surfacing welding. The detailed operational parameters were shown in Table 2.

The objective of the experimental process is to explore the influences of different inclinations of welding torch on the formation of weld bead and then seek the corresponding rules, so during the experimental process, apart from the inclination of welding torch, other operational parameters should be unchanged.

**Figure 2:**
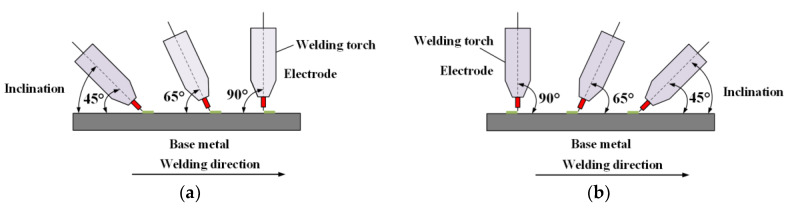
The presentation of two inclination modes. (**a**) Forward inclination angle, (**b**) backward inclination angle.

In this work, the inclination of welding torch denoted the angle between axis of the welding torch and horizontal line. According to different inclining directions, the inclinations have two modes: forward inclination mode and backward inclination mode. The forward inclination mode meant that the upper terminate of the welding torch was behind the lower terminate along the welding direction, and the opposite situation was the backward inclination mode. For these two modes, the corresponding angles were respectively forward inclination angle and backward inclination angle. Figure 2 showed the presentations of these two inclination angle modes.

In addition, during the experimental process, the welding torch was moving along the welding direction. For each experiment, the inclination angle was fixed and the torch only inclined to the welding direction and did not inclined to the left or right, in other word, the two terminates of the torch were traveling along the middle of the weld bead and each weld bead was symmetrical in this work.

According to above statements, corresponding experimental procedures were as follows:

1. The experiments were conducted by two arrays, one array for forward inclination mode and the other for backward inclination mode. In each array, the inclination angle was set between 45° and 90° with 5° interval, which had 10 experiments. Then, surface topography was examined to explore the characteristics.
2. During the experiments, three important geometry characteristic parameters, which were bead height, penetration and bead width, were seriously measured. For the bead width and bead height, 10 points with the same distances were picked up in each weld bead, then the average values were obtained. Then, the weld bead was cut and the corresponding metallographic specimen was obtained after a series of standard chemical processes, which included using Nital to etch the specimen, anhydrous alcohol to wash the specimen, and other corresponding operations, and then an industrial micro-camera was used to observe and measure the specimen. The penetration can be obtained using an industrial micro-camera; then, the average value of two penetrations can be confirmed as the final measurement value of the penetration. In addition, using the industrial micro-camera can obtain other values of bead width and bead height; then, the averages of preceding measurements and values using industrial micro-camera can be considered as the final measurement values of the bead width and bead height.
3. Curve fitting equations between inclination angles and geometry characteristic parameters were elaborated, and then corresponding verification experiments were conducted to validate the effectiveness and reliability of the curve fitting equations.
4. According to the experimental and corresponding analyses, the influence rules of the inclination of weld torch on the characteristics of weld bead were comprehensively induced.

## 3. Experimental Results and Analyses

Corresponding welding experiments were conducted. In this section, experimental results and corresponding analyses were presented.

### 3.1. The Effect of Forward Inclination Angle.

First, the forward inclination mode was employed; the surfaces and cross sections of corresponding 10 experimental results are shown in Table 3.

According to presentations in Table 3, the qualities of the welding beads improved with the increasing of the forward inclination angles. It can be noticed that the specimen Q1 had the worst surface appearance, the width of weld bead was not uniform, and undercut and arc restarting phenomena appeared several times; in addition, more spatters appeared, and the spatters were big and concentrated in the middle and the terminate of the weld bead. Additionally, the metal transfer process was unstable. The specimen Q2 also had a bad surface appearance, and the width and height of the weld bead were not uniform. Furthermore, between 12 cm and 13 cm in the figure, the width and height simultaneously decreased, and the undercut appeared with the arc restated in this position. In addition, the overall welding process was not successive and many spatters appeared. The weld bead of specimen Q3 was not straight, and the width and height were not uniform; additionally, the ripples between 10 cm and 16 cm were so rough. The width of weld bead of specimen Q4 suddenly decreased between 10 cm and 11 cm, and corresponding ripples were rough between 13 cm and 16 cm. The weld bead of specimen Q5 had bad surface appearance, and the width was not uniform; additionally, the bead was not straight. The weld bead of specimen Q6 was so straight, and the overall width was approximately uniform, but the height suddenly decreased between 13 cm and 14 cm, and the arc was badly terminated. The weld bead of specimen Q7 had good surface appearance and regular formation, and the width and height were uniform. The weld beads of specimen Q8 and Q9 had good surface appearance, the beads were straight enough, and the welding processes were successive; in addition, both of width and height of the beads were uniform. For the last specimen Q10, though the surface appearance was satisfied, the welding process was successive, and had uniform width and height; the bead was not straight, and some bending existed.

Additionally, according to cross sections in Table 3, from specimens Q1 to Q10, with the increasing of the inclination angles of the welding torch, the penetrations of the weld bead were also increasing.

### 3.2. The Effect of Backward Inclination Angle

Furthermore, the backward inclination mode was employed, and the corresponding 10 experimental results using backward inclination angles are shown in Table 4.

According to these 10 specimens using backward inclination angles, as the inclination angles increased, the formation qualities of weld beads also improved. In Table 4, the overall appearance of weld bead of specimen H1 was the worst, and the width and height were not uniform. In addition, the metal transfer process was unstable, large spatters appeared, some metal knobs gathered on the surface between 3 cm and 4 cm, and the ripples between 10 cm and 16 cm were so rough. The appearance of the weld bead of specimen H2 was still so bad, the metal transfer process was unstable, and the spatters appeared at two sides of the weld bead and surface of the bead. The width and height of the bead were suddenly decreased, which meant that undercut appeared, and then the arc restarted. The weld bead of specimen H3 was so straight, and the width were uniform, but some spatters appeared between 5 cm and 10 cm. The formation and width variation of the weld bead of specimen H4 were so regular, few spatters appeared, and the ripples between 7 cm and 16 cm were so dense and rough. The formation of weld bead of specimen H5 was not good, and the width and height of the bead between 10 cm and 13 cm were simultaneously decreased, which meant that the undercut appeared at this position, and the arc was restarted, as well as crater appeared at the terminate of the bead. The weld bead of specimen H6 had a high quality formation, the width and height were so regular, and however, the bead bended at the position of 10 cm, and the ripples between 7 cm and 16 cm were so rough. The appearance quality of weld bead of specimen H7 was so high, and the ripples between 12 cm and 16 cm were so rough; in addition, the width and height were suddenly decreased, and then the arc finished. The weld bead of specimen H8 was so straight, the height was so regular, and the width decreased at the position of 8 cm. At last the specimen H9 and H10 had weld beads with high quality, the formation was so regular and the bead was so straight. In addition, both of the width and height were so regular, and the overall welding process was successive. The metal transfer process was also stable, and few spatters appeared.

Moreover, as examining the cross sections in Table 4, it can be seen that from specimen H1 to H10 that the penetrations of the weld bead were also increasing. Hence, it can be known that no matter the forward or backward inclination mode, the penetrations of the weld bead were increasing as the increasing of the inclination angles. The detailed phenomena analyses will be conducted in next part.

According to the analyses in current and preceding part, when the inclination angles were above 70°, weld beads with satisfactory qualities can be obtained, no matter using forward or backward inclination mode. It can be concluded that the quality of the weld bead was relative to the inclination angle. 

### 3.3. The Effect of Inclination Angle on the Geometry Characteristics

**Figure 3:**
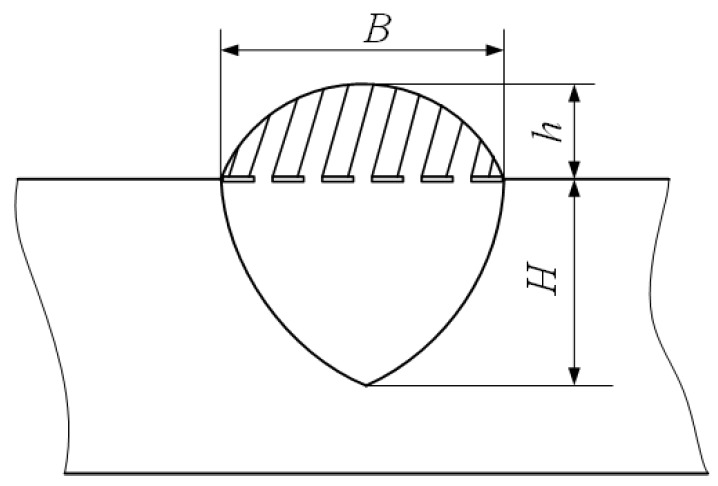
Three geometry characteristic parameters of the weld bead.

To further explore the influence rule of inclination of welding torch on the characteristics of weld bead, after examining the surfaces and cross sections of the weld bead, the detailed geometry characteristic parameters, such as bead width *B*, bead height *h* and penetration *H*, should also be accurately measured and then made corresponding analyses. These three geometry characteristic parameters were shown in Figure 3.

In this work, former weld beads obtained from forward inclination angles and backward inclination angles can be seriously measured to obtain the values of *B*, *h* and *H*, and then used the corresponding average values, which were obtained from selected 10 points with the same distance for bead width and bead height, and left and right measurement values by means of industrial camera for penetration, as stated in Section 2, the results were shown in Table 5 and Table 6. Apart from these three values, the ratio between penetration (*H*) and the thickness of the base metal (*S*), and the ratio between B and h, were also shown in these tables.

According to Table 5, the specimen Q1 had the smallest penetration, the melted liquid metal and base metal was not effectively joined, and the penetration was only 13.7% of the total thickness of the base metal; this proportion was so small that it could have seriously affected the mechanical performance of the joined metal components when it was used for joining two or more metal sheets. As the increasing of the inclination angles, the ratio *H*/*S* was also increasing, which meant that more liquid metal and base metal joined. In the specimen Q1, this proportion achieved 50%, and the corresponding proportion of specimen Q10 was 60.67%, while the corresponding penetration was proper. It can be noticed that when the inclination angles were between 45° and 90°, the ratios between *H* and *S* were increasing, the variation range of *H*/*S* was 43.67%, and the bead height coefficient, which was the fifth row of the Table and marked as *B/h*, had a relative stable value, which was about 2.7.

According to Table 6, as the same as that of forward inclination mode, the specimen H1 had the smallest penetration, and the corresponding *H*/*S* was 37.33%. This small penetration can affect the mechanical performance of the joined components. As the increasing of the inclination angles, the proportions of penetration in the thickness of base metal were also increasing. For the specimen H10, the corresponding *H*/*S* achieved 67.67%, and this ratio was proper. Under this circumstance, as the variation of backward inclination angles, the variation range of *H*/*S* was 30.34%, while the weld height coefficient *B/h* was about between 2.3 and 2.5, which meant that the *B/h* in the backward inclination mode was few smaller than that in the forward inclination mode, and the corresponding bead was so slim, which meant corresponding quality of weld bead was a little lower.

**Figure 4:**
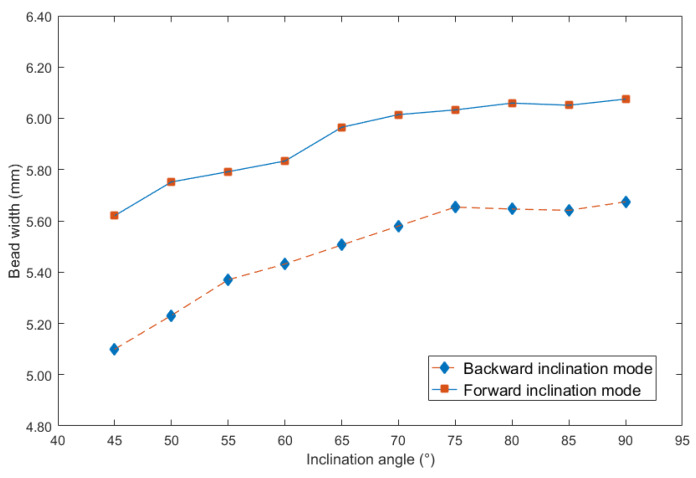
Curve between inclination angle and bead width.

**Figure 5:**
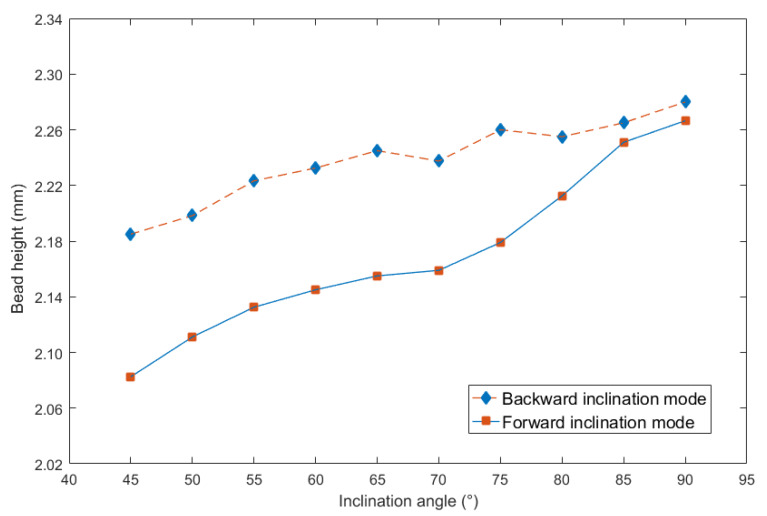
Curve between inclination angle and bead height.

**Figure 6:**
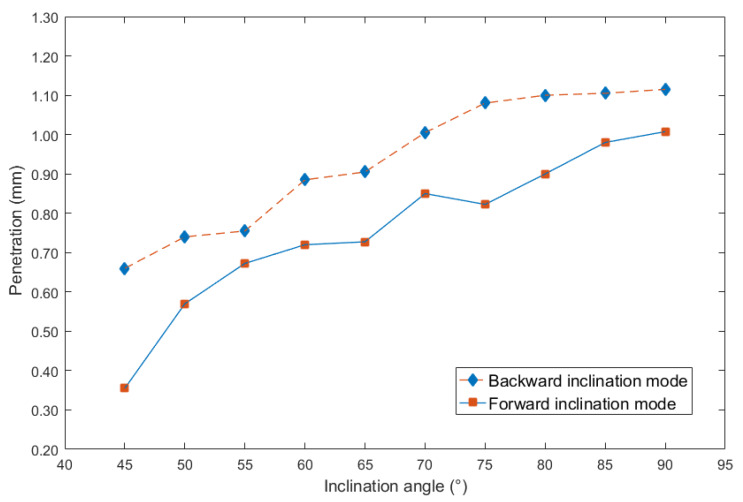
Curve between inclination angle and penetration.

To more clearly obtain the influence rules, and then compare the effects of different inclination angles on the weld bead formation, the curves between inclination angles and above three geometry characteristic parameters can be respectively shown in Figure 4, Figure 5 and Figure 6.

It can be seen from Figure 4 that no matter under forward and backward inclination modes, the bead width was increasing as the increasing of the inclination angles. However, when the inclination angles were the same, the bead width obtained from forward inclination mode was bigger than that from backward inclination mode. In addition, when the inclination angles were between 45° and 75°, the increasing speed under backward inclination mode was higher than that under forward inclination mode. Then, when the inclination angles were above 75°, the increasing speed under two modes was obviously slower than that below the angle, in other words, the slope of the curve was smaller when the inclination angles were beyond 75°.

It can be noticed from Figure 5 that the bead height was also increasing as the increasing of inclination angles, and when the inclination angles were the same, the bead height obtained from the backward inclination mode was bigger than that from the forward inclination mode, however, the increasing magnitude under the forward inclination mode was bigger than that under the backward inclination mode. When the inclination angles were between 45° and 85°, the bead heights under the two modes had obvious difference. However, after 85°, corresponding differences were smaller and smaller. It can be concluded that the effect of forward inclination mode on increasing bead height was larger than that of backward inclination mode.

According to Figure 6, the penetration was also increasing with the increasing of inclination angles. In addition, when the inclination angles were the same, the penetration obtained from the backward inclination mode was bigger than that from forward inclination mode, however, the increasing magnitude under the forward inclination mode was larger than that under the backward inclination mode. It can be concluded that under the forward inclination mode, the impact and excavation of the droplet on base metal was much stronger, which can induce larger penetration.

In addition, it can be noticed that as the inclination angle approached 90°, the curves in Figure 5 and Figure 6 were approaching, because at the inclination angle of 90°, the forward inclination mode and backward inclination mode should be the same. However, in Figure 4, the curves still had large differences, possibly because of some random errors due to the nozzle of the welding torch was not perpendicular to the plane of the terminate of the welding torch, also, there were other random and operational errors which may induce the inclination between actual electrical arc and the base metal not the same as that between welding torch and welding direction. Additionally, the experiments using forward inclination mode and backward inclination mode were individual conducted, the different measurements or other reasons induced the errors of the bead widths in this experiment. It can be concluded that for the weld width in Figure 4, the influence of the operational and instrumental errors on the measurements was larger than that of other two characteristic parameters, which were weld height and penetration.

## 4. Curve Fitting between Inclination Angles and Geometry Characteristic Parameters

In this section, to more conveniently and seriously explore the influence rule, based on the measurement data in the previous section, curve fitting equations between inclination angles and geometry characteristic parameters, such as bead width, penetration and bead height, can be conducted.

### 4.1. Curve Fitting Equation between Inclination Angle and Bead Width

**Figure 7:**
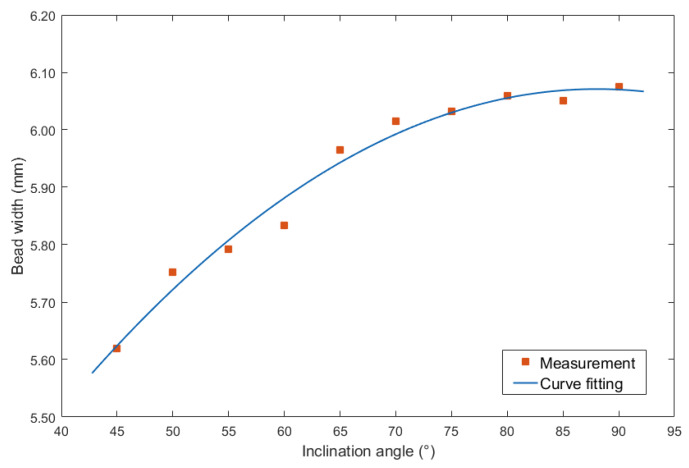
Curve fitting between forward inclination angle and bead width.

First, the bead width was considered. Figure 7 showed the curve fitting equation between inclination angle under forward inclination mode and bead width.

Second order polynomial equation was employed to obtain the mathematical description of the curve fitting equation, which was shown in Equation (1):

(1) $$y=-0.00024x^{2}+0.0425x+4.201$$

The largest errors of the curve fitting equation was 0.05 mm, and the corresponding mean squared error (MSE) was 0.0007. In addition, the R-squared (R^2^) was 0.9788, the Sum of Squares Due to Error (SSE) was 0.004799, and the Root mean squared error (RMSE) was 0.02618.

**Figure 8:**
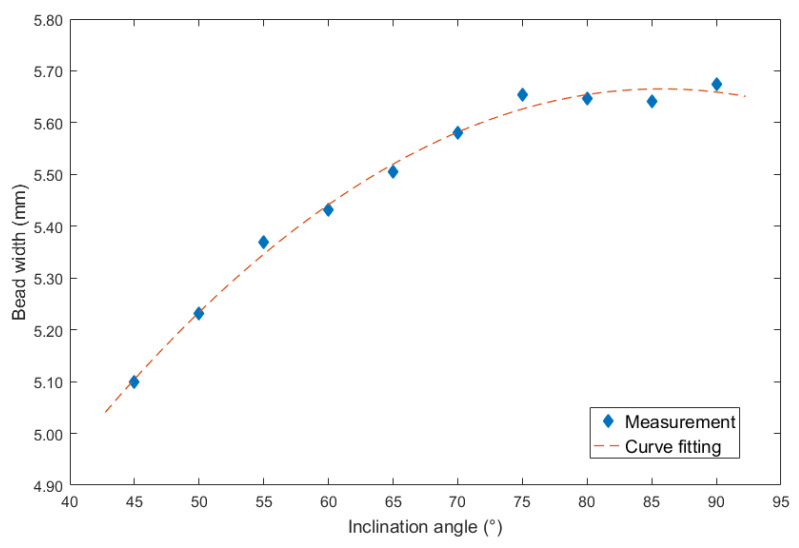
Curve fitting between backward inclination angle and bead width.

On the other hand, the curve fitting equation between inclination angle under backward inclination mode and bead width was shown in Figure 8. 

The corresponding second order polynomial equation was also employed, and the mathematical description was shown in Equation (2):

(2) $$y=-0.00034x^{2}+0.05795x+3.181$$

The largest errors of the curve fitting equation was 0.0024 mm, and the corresponding MSE was 0.0004. In addition, the R^2^ was 0.9908, the SSE was 0.002515, and the RMSE was 0.01895.

### 4.2. Curve Fitting Equation between Inclination Angle and Bead Height

**Figure 9:**
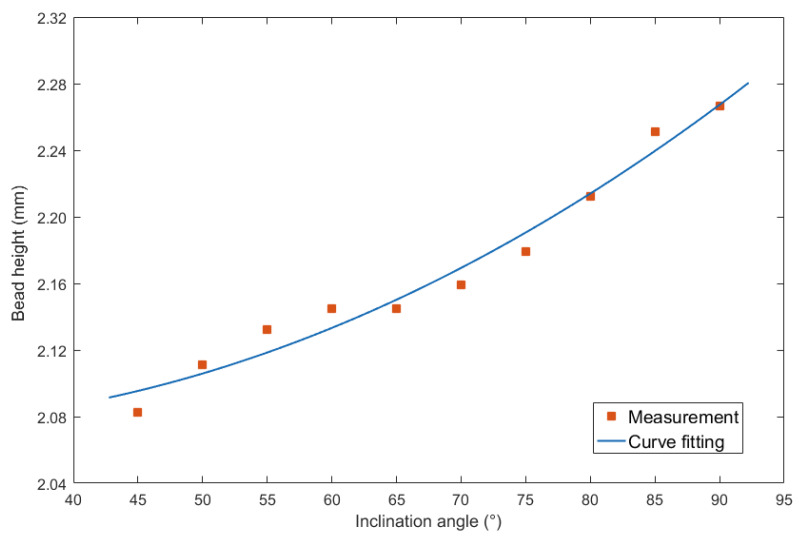
Curve fitting between forward inclination angle and bead height.

The last item was the bead height. For the inclination angles under forward inclination mode, corresponding curve fitting equation results were shown in Figure 9.

The result of using second order polynomial equation was shown as follows:

(3) $$y=0.000043x^{2}-0.00201x+2.099$$

The largest errors of the curve fitting was 0.015 mm, and the corresponding MSE was 0.0001. In addition, the R2 was 0.9706, the SSE was 0.0009287, and the RMSE was 0.01152.

**Figure 10:**
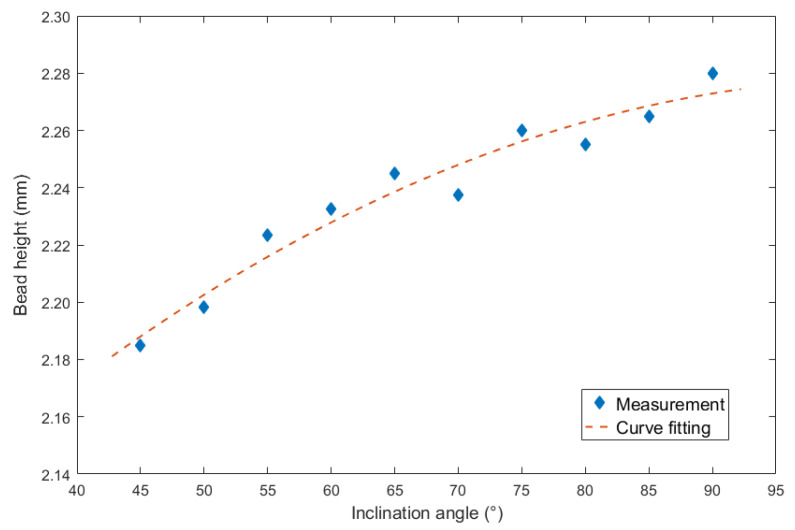
Curve fitting between backward inclination angle and bead height.

For the inclination angles under backward inclination mode, corresponding curve fitting figure was shown in Figure 10.

The result of mathematical description was as follows:

(4) $$y=-0.000026x^{2}+0.00535x+1.999$$

The largest errors of the curve fitting equation was 0.008 mm, and the corresponding MSE was 0.00006. In addition, the R^2^ was 0.9499, the SSE was 0.0003984, and the RMSE was 0.007544.

### 4.3. Curve Fitting Equation between Inclination Angle and Bead Width

**Figure 11:**
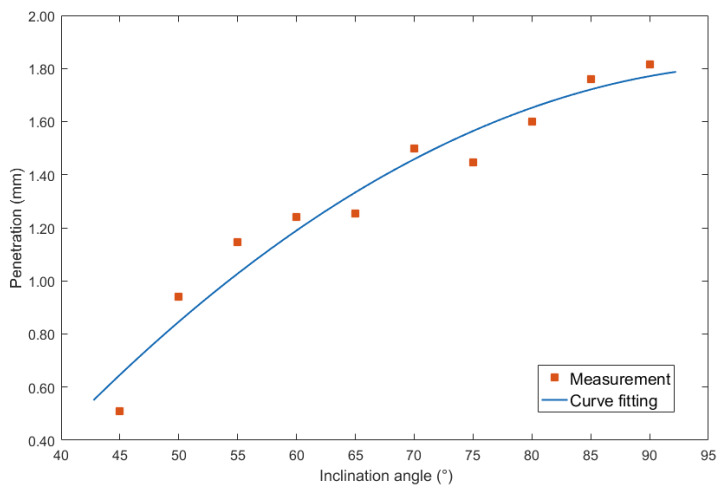
Curve fitting between forward inclination angle and penetration.

The second considered characteristic parameter was the penetration. Figure 11 showed the curve fitting between inclination angles under forward inclination mode and penetration.

Employing second order polynomial equation to mathematically described the relation as follows:

(5) $$y=-0.00037x^{2}+0.0755x-1.994$$

The largest errors of the curve fitting equation was 0.117 mm, and the corresponding MSE was 0.0103. In addition, the R^2^ was 0.9487, the SSE was 0.07213, and the RMSE was 0.1015.

**Figure 12:**
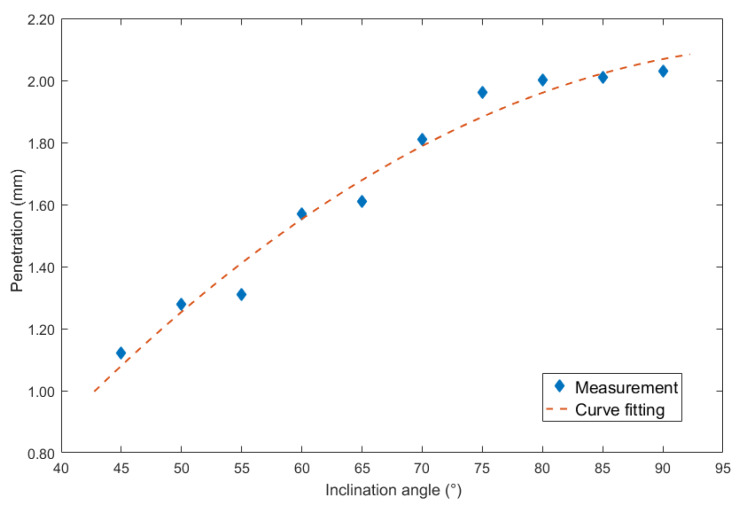
Curve fitting between backward inclination angle and penetration.

Then for the inclination angles under backward inclination mode, corresponding curve fitting figure was shown in Figure 12.

The second order polynomial equation was used to mathematically describe the relation as follows:

(6) $$y=-0.00032x^{2}+0.0649x-1.197$$

The largest errors of the curve fitting equation was 0.101 mm, and the corresponding MSE was 0.0039. In addition, the R^2^ was 0.9742, the SSE was 0.02724, and the RMSE was 0.06238.

## 5. Experimental Verification of the Curve Fittings

### 5.1. Verified Experiments

In Section 4, the influence rules of different modes and values of inclination angles on the weld bead characteristics during pulsed-GMAW process based on the robotic operation was seriously explored, and then the corresponding curve fitting equations between inclination angles under different modes and geometry characteristic parameters of the weld bead were also obtained. In this section, corresponding verification experiments were conducted to verify the reliability and effectiveness of above curve fitting equations. Apart from the inclination angles, other operational parameters referred to those mentioned in Section 2. In this work, Q11 was marked when the forward inclination angle was 68° and H11 was marked when the backward inclination angle was 68°. The surfaces and cross sections of the weld bead were shown in Table 7.

### 5.2. Verification Analyses

Then the errors between actual measurements and curve fitting equations were shown in Table 8, in this table, all the errors were recorded using absolute format.

According to the verification results, it can be noticed that the curve fitting errors from the inclination angle under backward mode were larger than those under forward mode. Among three geometry characteristic parameters of weld bead, the largest error between curve fitting equations and actual measurements occurred at the penetration. For the penetration, under the forward inclination mode, the largest error was 0.10 mm, which was 7.52% of the actual measurement; while under the backward inclination mode, the largest error was 0.15 mm, which was 9.43% of the actual measurement. The results showed that the curve fitting equations between inclination angles and geometry characteristic parameters of weld bead can be used to predict the geometry characteristic parameters, and only small errors were induced. Hence, it can be employed to instruct the improvement of the GMAW process, or control the geometry characteristic parameters of the output weld bead.

## 6. Conclusions and Future Work

The objective of this work is to explore the influence rules of inclination of welding torch on the weld bead characteristics during pulsed-GMAW process based on robotic operation. In the work, the surface topography and geometry characteristic parameters were the outputs of the welding operational process, because they can reflect the welding quality and the stability of the welding process. The inclination angle of the welding torch can be catalogued into forward inclination mode and backward inclination mode. Under these two modes, 10 arrays experiments which used 5° interval were conducted, and then the influences of the inclination angles on the surface topography and cross sections can be induced by means of corresponding measurements and analyses. Then according to the serious measurement combining the mathematical analyses, curve fitting equations using second order polynomial formats between inclination angles and bead width, penetration and bead height. After examining and analyzing the results, it can be noticed that the largest errors between the measurements and curve fitting equations under the forward inclination mode were 0.01 mm, 0.07 mm and 0.10 mm, while under the backward inclination mode, the corresponding errors were 0.06 mm, 0.14 mm and 0.15 mm. After a series of analyses and corresponding experiment verifications, following conclusions can be driven:

1. When other operational conditions and parameters were unchanged, only changed the values of inclination angles of welding torch, the formation and characteristic parameters of weld bead were significantly affected by the modes and values of inclination angles. Increasing the inclination angles can improve the quality of the weld bead. No matter under the forward inclination mode or backward inclination mode, when the inclination angle was above 70°, the weld bead with high quality can be obtained. In addition, the bead height coefficient under forward inclination mode was larger than that under backward inclination mode, as well as the effect of inclination angle variation on the penetration was also higher. The weld bead under the backward inclination mode was much slimmer, which corresponded to weld bead with low quality. Furthermore, the variation range under the backward inclination mode was smaller than that under forward inclination mode.
2. The inclination angles can affect the geometry characteristic parameters of the weld bead, and all the parameters can increase as increasing of the inclination angles. When compared the two modes, which were forward inclination mode and backward inclination mode, the bead width under the former mode was larger than that under the latter mode when the inclination angles were the same, however, for other two parameters, which were penetration and bead height, corresponding conclusions were opposite.
3. Corresponding curve fitting equations between inclination angles and geometry characteristics parameters were obtained to induce the influence rules, then verification experiments were conducted and the results showed that the influence of inclination angles on the geometry characteristics parameters can be mathematically described using a series of second order polynomial equations, and all the errors of curve fitting were below 10%. Hence, the equations can be employed to predict the geometry characteristic parameters of weld bead during the process.

## Figures and Tables

**Table 1:** Chemical composition (mass fraction/%) of base metal and welding wire.

Materials	C	Si	Mn	Cr	Ni	S	P	N	Mo
304 Stainless Steel	≤0.08	≤1	≤2	18~20	8~10.5	≤0.03	≤0.03	≤0.1	–
316L Stainless Steel	≤0.03	≤1	≤2	16~18	10~14	≤0.03	≤0.045	–	2~3

**Table 2:** The operational parameters of the welding experiment.

Average Arc Voltage (V)	Average Current (A)	Welding Speed (cm/min)	Wire Feed Speed (m/s)
19.6	80	30	1.2

**Table 3:** The surfaces and cross sections of the weld bead under forward inclination mode.

No.	Angle	Surface Topography	Cross Section
Q1	45°	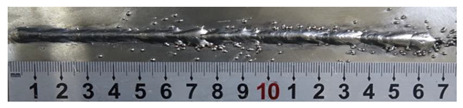	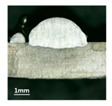
Q2	50°	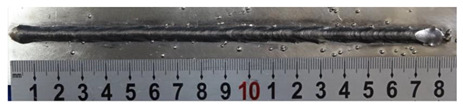	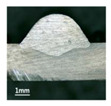
Q3	55°	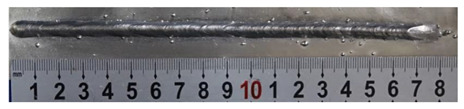	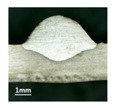
Q4	60°	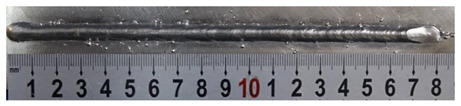	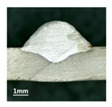
Q5	65°	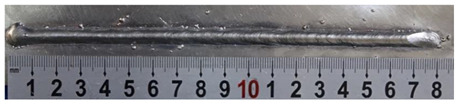	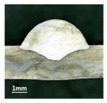
Q6	70°	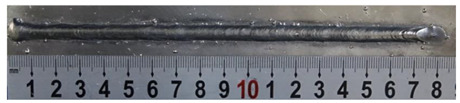	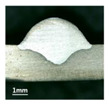
Q7	75°	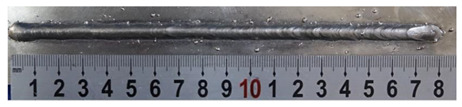	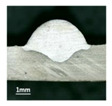
Q8	80°	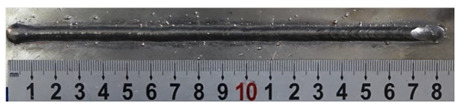	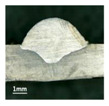
Q9	85°	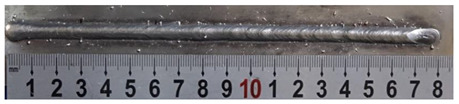	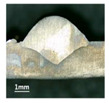
Q10	90°	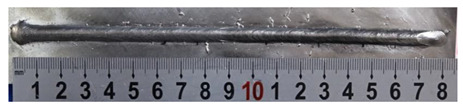	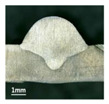

**Table 4:** The surfaces and cross sections of the weld bead under backward inclination mode.

No.	Angle	Surface Topography	Cross Section
H1	45°	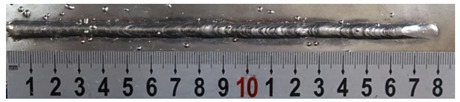	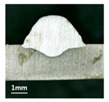
H2	50°	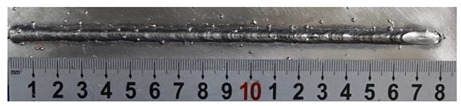	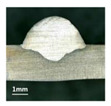
H3	55°	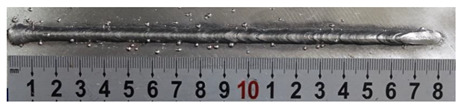	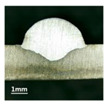
H4	60°	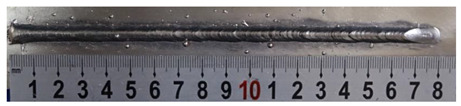	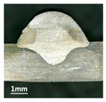
H5	65°	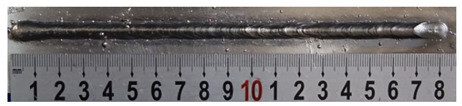	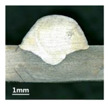
H6	70°	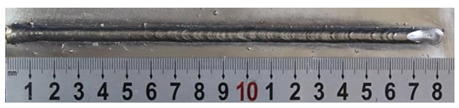	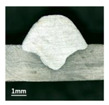
H7	75°	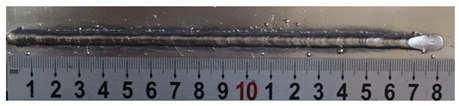	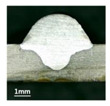
H8	80°	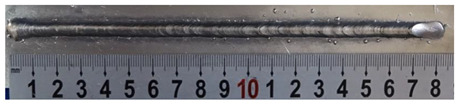	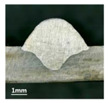
H9	85°	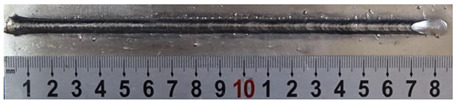	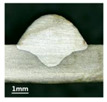
H10	90°	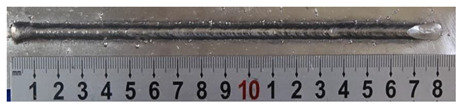	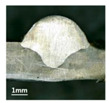

**Table 5:** Measurement of geometry characteristic parameters under forward inclination angles.

Parameter	Q1	Q2	Q3	Q4	Q5	Q6	Q7	Q8	Q9	Q10
*B* (mm)	5.62	5.75	5.79	5.83	5.97	6.01	6.03	6.06	6.05	6.08
*h* (mm)	2.08	2.11	2.13	2.15	2.15	2.16	2.18	2.21	2.25	2.27
*H* (mm)	0.51	0.94	1.23	1.24	1.26	1.52	1.45	1.60	1.76	1.82
*H*/*S* (%)	17.00	31.33	41.00	41.33	42.00	50.67	48.33	53.33	58.67	60.67
*B*/*h*	2.70	2.73	2.72	2.71	2.78	2.78	2.77	2.74	2.69	2.68

**Table 6:** Measurement of geometry characteristic parameters under backward inclination angles.

Parameter	H1	H2	H3	H4	H5	H6	H7	H8	H9	H10
*B* (mm)	5.10	5.23	5.37	5.43	5.51	5.58	5.65	5.65	5.64	5.67
*h* (mm)	2.19	2.20	2.22	2.23	2.25	2.24	2.26	2.26	2.27	2.28
*H* (mm)	1.12	1.28	1.31	1.57	1.61	1.81	1.96	2.00	2.01	2.03
*H*/*S* (%)	37.33	42.67	43.67	52.33	53.67	60.33	65.33	66.67	67.00	67.67
*B*/*h* (%)	2.38	2.38	2.42	2.43	2.45	2.49	2.50	2.50	2.48	2.49

**Table 7:** The surfaces and cross sections of the weld bead of the varication experiments.

No.	Angle	Surface Topography	Cross Section
Q11	68°	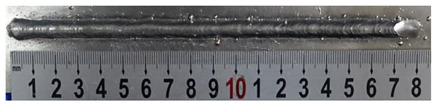	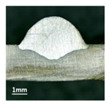
H11	68°	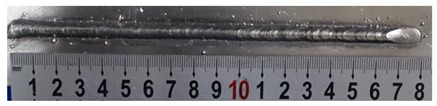	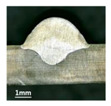

**Table 8:** Verification results (\mm).

No.	Parameters	Measurement	Results from Curve Fitting	Error	Error Percent (%)
Q11	*B*	5.99	5.98	0.01	0.17
	*h*	2.23	2.16	0.07	3.14
	*H*	1.33	1.43	0.10	7.52
H11	*B*	5.61	5.55	0.06	1.07
	*h*	2.38	2.24	0.14	5.88
	*H*	1.59	1.74	0.15	9.43
